# The perspectives of Lagos faith-institution leaders on health, hypertension, and faith-based hypertension interventions: a qualitative interview study

**DOI:** 10.3389/fpubh.2026.1925205

**Published:** 2026-09-09

**Authors:** Abayomi Sanusi, Helen Elsey, Su Golder, Olamide Todowede

**Affiliations:** 1 Department of Health Sciences, University of York, York, United Kingdom; 2 Health Services Management Centre, University of Birmingham, Birmingham, United Kingdom

**Keywords:** community, faith institutions, faith leaders, health protection and promotion, hypertension, Lagos, Sub-Saharan Africa

## Abstract

**Introduction:**

Hypertension remains a leading contributor to morbidity and mortality in Nigeria, where health system resources are constrained. Faith institutions wield considerable community influence and have supported previous health initiatives; however, their potential role in hypertension prevention and management remains critically understudied. This study explored the perspectives of faith-institution leaders in Lagos on health, hypertension, and the feasibility of faith-based interventions.

**Methods:**

We purposively sampled eight diverse faith leaders representing Sunni Islam, Pentecostal Christianity, African Traditional Religion, Ahmadiyya Islam, Judaism, and Catholicism in Lagos. Data were collected via remote semi-structured interviews and analysed using a purely inductive thematic analysis approach, involving open line-by-line coding, constant comparison of codes to refine categories, and iterative derivation of descriptive and analytical themes. Reporting adheres to the COREQ checklist.

**Results:**

Four overarching themes emerged: (1) health system deficiencies—encompassing inadequate infrastructure, socioeconomic pressures, and waning public trust—were perceived as a fundamental impediment to hypertension control; (2) faith leaders observed widespread poor hypertension knowledge and poverty-driven health-seeking behaviors leading to preventable deaths; (3) their institutions, although underutilized, possess trusted physical spaces and community networks suitable for health promotion; and (4) there was strong willingness among leaders to collaborate with government and NGOs to deliver congregation-tailored screening, education, and referral interventions.

**Discussion:**

Faith leaders in Lagos demonstrate a sophisticated understanding of the societal determinants of hypertension and express clear willingness to act as health assets. Their institutions represent a promising, yet currently underexploited, platform for integrating community-based NCD prevention into urban health systems. These findings offer transferable insights for leveraging faith-based networks in low-resource settings, contributing to the broader agenda of strengthening primary healthcare access (SDG 3.8) and reducing NCD burden in sub-Saharan Africa. To address the hypertension epidemic in Lagos, Nigeria, and beyond, leveraging faith institutions’ unique influence, authority and assets requires recognising leaders’ conditional willingness as a rational defence of their legitimacy—necessitating genuine, reciprocal partnerships that respect them as equal partners, not convenient access points.

## 1 Introduction

Hypertension is a leading cause of cardiovascular death, with low- and middle-income countries bearing a disproportionate burden (1, 2). In Nigeria, prevalence is 30%–40%, but awareness, treatment, and control are low (3, 4). The health system faces inadequate infrastructure, funding shortages, professional emigration, and limited access to care — especially in urban informal settlements (5–7).

Lagos, Nigeria’s largest city (population >20 million), exemplifies these challenges. Rapid urbanisation, poverty, and overstretched public health services created an environment where non-communicable diseases, especially hypertension, are increasingly prevalent but poorly managed (8, 9). Emergency presentations for hypertension-related complications remain common, indicating failures in early detection and primary prevention (10, 11).

In many low-resource settings, faith institutions served as trusted community assets for health promotion, filling gaps left by under-resourced health systems (12, 13). Faith-leaders exercise considerable social, cultural, and political influence in Nigeria, where religious participation is near-universal and deeply embedded in daily life (14, 15). Faith institutions contributed to health initiatives addressing HIV/AIDS, maternal and child health, and immunisation (16–18). Evidence on their potential role in hypertension prevention and management in Nigeria remains scarce.

Understanding faith institution leaders’ (that is, faith leaders’) perspectives is essential for designing acceptable, feasible, and contextually appropriate hypertension interventions. Faith-leaders act as gatekeepers and influencers within their congregations; their support is critical to any faith-placed health initiative (19, 20). Their legitimacy as religious and community authorities is therefore fundamental to understanding how partnerships might be negotiated and sustained.

This study explored Lagos faith leaders’ perspectives on health, hypertension, and faith institution facilitated hypertension intervention, guided by four questions: “How do they view faith institution users’ hypertension experiences and their own?”; “How do Faith-leaders view hypertension awareness in society?”; “What are their perspectives on healthcare availability to mitigate hypertension?”; and “What are their views on the potential roles of their institutions to mitigate hypertension?”

## 2 Materials and methods

### 2.1 Study design

The exploratory research questions required approaches suitable for eliciting rich, in-depth, and potentially complex ideas. The design was qualitative, using semi-structured interviews to explore the perspectives needed to meet the stated objectives.

### 2.2 Conceptual framing

Conceptualising blood pressure and hypertension as a disease process is far from straightforward, and addressing hypertension has grown in complexity (21–24). The understanding of and reaction to hypertension by religious people can be complex, philosophical, varied, and emotive (25–27). An interview guide was designed to focus participants on the research questions (Table 1). The guide adapted phenomenological interviewing principles (28, 29) and a structure for contextualising, apprehending, clarifying, and extracting informants’ deep thoughts on a particular situation, lived experience, or phenomenon (30).

**Table 1:** Interview guide.

Question domains	Step 1: Establishing informant’s experiences/opinions/perception	Step 2: Constructing informant’s experiences/opinions/perceptions	Step 3: Meaning of informant’s experiences/opinions/perceptions	Step 4: Reflection on the meaning of the experiences/opinions/perceptions
Hypertension experience	Please tell me your personal views about hypertension (high blood pressure) as a health issue. [A definition and explanation provided if needed]. Can you use particular examples from yourself and users of your institution that illustrate this view/opinion?	Please tell me about any experience or situation you or any user of your institution has had in the past with hypertension as a health issue.	In your personal view, what is the meaning or interpretation of these experiences / situations?	What are the implications of this view? (Alternatively: what changes would have meant these views were completely different or better?)
Hypertension awareness	In society, do people know about hypertension (i.e., have awareness about what it is, or whether or not they have it). Your personal views about this awareness situation?	If we were to apply your views / thoughts to users of your institution who may not have enough awareness: how would you describe the awareness situation?	In your personal view, what is the meaning or interpretation of the awareness situation?	What are the implications of this view about awareness? (Alternatively: what realities would have meant your view(s) about hypertension awareness was completely different?)
Local healthcare provision	Please tell me your personal opinion or observation about the healthcare available to deal with hypertension as a health issue [for the users of your institution].	How would you describe the situation about the availability of healthcare to deal with hypertension?	In your personal view, what is the meaning or interpretation of that described situation of healthcare availability for hypertension?	What are the implications / importance of this view? (Alternatively: what would have made your views completely different from what they are now?)
Capabilities of informant’s own institution	Given suitable conditions some faith institutions may have capabilities to or be willing to participate in activities toward reducing hypertension of their users. What capabilities do yours have? What kind of activities might your institution be able to participate in toward this end?	What are your opinions and thoughts about your institution to participating in activities that lead to reduction of hypertension in participants?	In your personal view, what is the meaning or interpretation of these opinions and thoughts?	What are the implications / importance of this view? (How could these views be actualised, or how could these be made to contribute to reduction of the frequency of blood pressure as a health issue?)

### 2.3 Sampling

Faith institutions approached were religious non-profit organisations that practised collective worship and had an ethos of voluntarily providing services to their networks and wider society when able.

The study’s narrow, well-defined aims could be addressed by a small number of informants with specific, relevant experiences. The final sample of eight participants, though limited by recruitment challenges, provided sufficiently rich and relevant information for these aims. Consistent with the concept of information power (31), the high specificity of the sample—comprising senior leaders from diverse religious traditions—and the dense data obtained from each interview support the adequacy of this sample size.

A purposive sampling strategy ensured diversity in Faith-leadership. This involved recruiting at least two leaders from distinct intra-religious traditions within Islam and Christianity, and one leader from outside those traditions (e.g., African Traditional Religion or Judaism). In Lagos, this represented a cross between a convenience sample (participants available) and a maximum variation sample (incorporating the breadth of faiths practised by most Lagosians).

The recruitment period ran from 17/08/2021 to 17/02/2022. The manuscript was subsequently prepared for submission in 2026. Recruitment continued until data saturation was achieved, defined as the point at which no new themes or codes emerged. Saturation was assessed iteratively during analysis. No new open codes emerged after the fifth transcript was coded.

Internet searches (Google, Duckduckgo, Bing, Yahoo), social media searches (primarily Facebook, Twitter/X, Instagram), local networks, diaspora contacts, and telephone were used to identify and contact institutions, organisations, charities, and leaders from Nigerian and Nigeria-based Christianity, Islam, Judaism, African Traditional Religion, Buddhism, and Hinduism. The search was repeated for organisations and institutions outside Nigeria with religious or denominational ties to Nigeria. National and state faith umbrella organisations (hierarchical denominational and interdenominational religious bodies) were also contacted. Using the local and international contacts, websites, social media handles, and phone numbers obtained, invitations were made to over 91 institutions. Responses were followed up with pre-interview questionnaires. From the 91 faith institutions contacted, 8 faith leaders ultimately participated.

Non-participation among Buddhist and Hindu leaders—whose decisions were respected given the voluntary nature of participation—likely reflects their minor demographic presence in Lagos, which is predominantly Muslim and Christian. Recruitment involved multiple meetings with gatekeepers, during which institutions screened the researcher (AS) through their internal processes—documented in field notes. Concepts from Weber’s theory of religious authority also informed recruitment. Applying the distinction between traditional, rational-legal, and charismatic authority helped navigate the multiple layers of access—gatekeepers then leaders. This pragmatic use of theory, alongside clinical background and transparency about the study’s aims, may have facilitated trust and engagement. Gatekeepers expressed initial interest for their leaders to participate, but these did not materialise due to late-stage withdrawals, internal bureaucracy, logistical challenges, or unexplained reasons.

This recruitment pattern may have introduced gatekeeper or self-selection bias toward leaders already favourably disposed to health engagement, potentially shaping the perspectives captured.

A faith leader was defined as any individual (any race, gender, educational background) who, on any basis (full-time, part-time, with or without remuneration), through and on behalf of any Lagos-based faith institution, delivers any combination of administrative, religious, educational, worship, or charitable duties to users, and can represent the institution publicly. The most senior leaders were targeted because they hold executive powers over institutional participation in interventions (32, 33) and also deliver or supervise interventions within faith institutions (34, 35). Their delegated replacements were accepted; no seniority level was excluded. The only inclusion criterion was voluntary participation. The only exclusion criterion was self-exclusion (non-response or withdrawal at any stage).

### 2.4 Data collection

The interview guide facilitated thinking in four steps: establishing experiences/opinions/perceptions; constructing those experiences; deciding their meaning; and reflecting on their meaning. Face-to-face interviews were not possible due to the Covid-19 pandemic. Data were collected using Zoom, telephone calls, and WhatsApp audio/video calls. Each interview lasted between 45 and 60 min. These complementary modalities minimised interruptions from technical difficulties (e.g., equipment, internet, or phone signal failures). Data were stored securely on a university server. All interviews were conducted by AS. The research team comprised AS (male), HE (female), SG (female), and OT (female).

### 2.5 Conduct of the interviews

Interviews in English used open-ended non-directive questions, capturing realities as Faith-leaders experienced and perceived them: their thoughts, feelings, and interpretations of hypertension lived experiences. The interviews were semi-structured, guided by the interview guide rather than a fully scripted verbatim format. Attention focused on non-interference with interviewees’ flow of thought — straightforward unless technical difficulties interrupted. Non-interference was easier than face-to-face. To minimise lag effects from remote communications, a brief pause followed each sentence. Probes included: “could you say more about that…?”, “do you have any examples in mind?”, “by that you mean…?”. Interruptions occurred only for clarifications, interviewee questions, or audio/visual technical issues. A reflexivity journal documented reflections immediately after each interview. Interviews were conducted by AS (MD, MSc, MPH), a doctoral researcher formally trained in qualitative research methods. HE, SG, and OT hold doctoral degrees (PhD), worked in research and academic roles and, in addition to their supervisory roles, contributed to the study design, methodology, analysis, and manuscript review. AS had prior experience conducting qualitative interviews and worked as a medical doctor internationally, including in the United Kingdom. No prior relationship existed between the researcher and any participant before the study commenced. Participants were informed that the researcher was a doctoral researcher at the University of York and that the study was part of his doctoral research. His clinical background and interest in hypertension were shared with participants during the informed consent process to provide transparency about his positionality. No other individuals were present during the interviews besides the participant and the researcher.

### 2.6 Generation of the transcripts

The researcher familiarised with the data through repeated listening and watching of each interview, then verbatim transcription. Two native Nigerian English speakers (proficient in professional English) conducted a language consultation: clarifying selected phrases, words, unique usages, and expressions to eliminate ambiguities.

### 2.7 Analysis

The visual element of interviews engaged and connected with Faith-leaders but was not used in analysis. Theme emergence was entirely data-grounded. Inductive analysis (iterative derivation of open/closed codes, descriptive themes, analytical themes, and their interactions) was conducted separately for each research question domain. Each transcript was read multiple times to familiarise further with the data. A purely inductive, bottom-up approach ensured ideas emerged strictly from the data, without predefined measures or *a priori* models (36–38). The methodological orientation was thematic analysis, following the principles of Thomas (36) and Bevan (37), which was pragmatically chosen as the most suitable approach to achieve the research objectives. The analysis prioritised patterns grounded in participants’ accounts over pre-existing frameworks, using manual coding in Microsoft Word. While initial coding and theme generation were inductively derived from participant narratives, the subsequent interpretive phase drew on Max Weber’s theory of religious authority (39) to conceptualise emergent patterns concerning leaders’ institutional legitimacy and partnership conditions. Respondents’ discourse preferences influenced the analysis. Respondents preferred evocative, lengthy narratives to illustrate responses and express ideas, painstakingly elaborating on surrounding issues before revealing answers. Therefore, lengthy, often emotive quotations have been kept intact in the reported findings and analyses, with relevant sections available for readers’ focus. All quotations are labelled with participant identifiers (Respondent 1–8), which can be cross-referenced with Table 2.

**Table 2:** Characteristics of faith leaders and their institutions.

Faith institution leader					Faith leaders’ local faith institution		
Participant	Gender, Age bracket	^¶^Hierarchy of leadership	Self-described profile	*Localisation, estimation of influence	Religion	Congregation size	**Localisation, estimation of influence
1	Male 50 to 70 years	Imam. Main leader.	Lagos-born passionate local community leader with academic training in politics and familiarity with the workings of local governments	Local and State level influence (past social and policy work at level)	Sunni Islam	Medium	Local community influence. Uncertain State level influence.
2	Male 50 to 70 years	Pastor. Main leader. Regional administrative leadership within denomination.	Pastor with decades of experience, ranking high in seniority within denominational hierarchy, and with good national connections	Local and State level influence (intra-Faith-leadership work at level)	Pentecostal Christianity	Medium	Local community influence. Uncertain State level influence
3	Male 50 to 70 years	Priest. Main leader at shrine and congregation	University educated community worker who facilitates networking between national and international supporters of his religion. Passionate about improving the lives of the urban poor, rural dwellers and underserved populations	Local, State, Regional and National level influence (leadership, policy and development work at level)	African Traditional Religion (ATR)	^#^Small	Local community, State, Regional and National influence
4	Male 50 to 70 years	Pastor. Main leader. Leadership responsibilities across several parishes in a subdivision of Lagos	A well-travelled information technology professional with international exposure within his faith, that believes education is the means to solving many societal ills	Local, State and Regional level influence (intra-Faith-leadership work at level)	Pentecostal Christianity	Medium	Local community, State, Regional and National influence
5	Male 30 to 49 years	Imam. Main leader.	Well-connected private sector executive, influential through charitable community projects	Local, State, Regional and National level influence (charity, administration and policy work at level)	Ahmadiyya movement Islam	Medium	Local community, State, Regional and National influence
6	Female 30 to 49 years	Priestess. Main leader.	Passionate about educating and improving the lives of uneducated, illiterate, and underserved populations	Local and State level influence (charity, publicity and education work at level)	African Traditional Religion (ATR)	Small	Local community and State
7	Male 30 to 49 years	Rabbi. Main leader.	Lives and works within his local community, is familiar and comfortable working with local community leaders on local projects	Local community level influence and work (National level intra-faith work)	Judaism	Small	Local community, Sub-State level influence. State intra-faith level influence.
8	Male 30 to 49 years	Priest. Main leader.	Heavily involved in community work and philanthropic programmes targeting the poor and underprivileged	Local community, State level influence (charitable work at this level)	Catholic Christianity	Large	Local community and State.

^¶^Hierarchy of leadership denotes the seniority of individuals as the main leader of their faith institution parish / congregation. Position below main leader, or delegated by the main leader are denoted ‘sub-leader’. Descriptions are added to highlight wider leadership activities or roles previously or currently held. *Localisation and estimation of individual faith leader influence denotes individual leader’s declared current or past activities within their capacity as a faith leader either on behalf of their individual parish or network of parishes within the same denomination. **Localisation and estimation of individual faith institution influence denotes the declared provision of social or charitable services by individual parishes to congregation of the parishes or the local community; or declaration of such services individual parishes had contributed to the wider community on as part of a denomination. Parish / congregation size: Number of people registered as permanent members of the parish. Small = up to 100 at a time, Medium = over a hundred but fewer than a thousand, Large = at least a thousand congregants or worshippers. ^#^Small = African Traditional Religion congregations no longer follow a model of regular large collective worship or gathering. Intra-faith denotes declaration of work, activities or influence as relating to a single religious faith, rather than across different religious faiths. Regional denotes a description across at least two states including Lagos within the South-Western geopolitical zone of Nigeria that includes Lagos. National denotes a description across several states over the six geopolitical zones in Nigeria.

#### 2.7.1 Derivation of open and closed codes

The coding strategy stayed close to the data using: values coding (capturing informants’ beliefs/attitudes); in-vivo coding (using informants’ own words); and narrative coding (incorporating aspects of informants’ stories) (40). Manual (Microsoft Word) open line-by-line coding was undertaken for each question/analysis domain, generating open codes for each sentence — each code describing one sentence’s meaning (37). Iterative constant comparison of all open codes merged similar codes and reassigned redundant ones. Final open codes were verified by matching to the data. Closed codes were derived by iteratively grouping open codes into overarching categories based on collective meanings of categorised final open codes. Iterative constant comparison of closed codes with transcript data ensured continued matching (37). All coding was undertaken by AS. The coding process was reviewed and discussed regularly with HE and SG to refine code definitions and ensure interpretive consistency.

#### 2.7.2 Derivation of the descriptive and analytical themes

Descriptive themes were generated by organising closed codes into categories based on the collective, overarching meaning of their content and how those meanings address the question domain. Iterative constant comparison of descriptive themes with transcript data examined how they address or answer the question (37). Ideas within descriptive themes were categorised by how they relate together to address the analysis domain’s question. These grouped descriptive themes together address the overarching domain question and formed the analytical themes. Constant comparison of ideas in descriptive themes to transcript data ensured all data were placed in a descriptive theme category. Analytical themes were exhaustive and addressed or reflected the domain question (37). Following the derivation of themes, the team reviewed the thematic structure to ensure that findings were consistent with the raw data. Discrepancies in interpretation were resolved through discussion and consensus.

#### 2.7.3 Derivation of the links and interactions

All interview quotes within each theme were collected to examine ideas within descriptive themes and explore their links, relationships, and interactions across three stages: re-examining quotes from ‘establishing the understanding’; ‘constructing the understanding’; and ‘reflecting upon the meaning and understanding’. Throughout this process, the field notes and reflections systematically collected by AS during and immediately after interviews informed the interpretation of data and contributed to the contextual understanding of participants’ narratives. This process finalised the themes (37).

### 2.8 Reflexivity

Faith-leaders’ high regard and societal importance were highlighted by multiple meetings, interviews, and committee opinions negotiated to gain access. Gatekeepers’ communication and negotiation skills suggested they often had equivalent or higher education than the leaders they controlled access to. This may indicate that educational qualifications, training, or similar refinement are not necessarily the most essential characteristics of faith leaders. It may also simply reflect how well faith-leaders are protected by protocol and gatekeepers.

One leader’s harsh words about the deleterious effect of lacking formal education on the uneducated poor and many uneducated wealthy could be perceived as uncharitable and potentially prejudicial. However, the context of their charitable activities toward the poor and uneducated did not suggest serious prejudice.

All leaders displayed caution and adeptness in not directly framing health system failures as political phenomena or rationales to criticise the government or politicians, but rather as health system issues with room for interpretation and improvement. After each interview, leaders were invited to ask and answer any pertinent questions not already covered. They all summarised and reinforced their earlier points.

The researcher took the pragmatic view that Faith-leaders’ perspectives and values could be creatively applied to address Nigeria’s complex hypertension problem. He has religious faith and longstanding interactions with diverse-faith research and healthcare professionals. His assumption that faith institutions are potential intervention assets is based on observing that religious teachings and faith-based networking generally promote physical and mental health. The researcher observes that faith institutions function as basic units of Nigerian society, similar to families.

Clinical practice familiarity with how hypertension complicates acute and chronic illnesses and causes worse outcomes contributed to his curiosity. Understanding the juxtaposition of increasing hypertension frequency in Nigeria and escalating healthcare workforce emigration positions the researcher as an outsider with useful insider knowledge. He acknowledges that his pragmatist outlook, religious faith, professional background, and perception of the contradiction between accelerating workforce emigration and escalating hypertension frequency potentially impact his role as a research tool. These, to his awareness, have not directly influenced data collection or analysis but were influential in designing the research questions. Hence, the described methodology (sampling, interviews, analyses) was implemented to mitigate potential biases.

The interviewer’s clinical background may have shaped the focus on biomedical aspects of hypertension. To mitigate this, the interview guide (Table 1) was designed to be open-ended, with probes limited to clarifications rather than directing content. During coding and analysis, AS’s clinical perspective was bracketed through team discussions (HE, SG) that challenged assumptions. No coding was finalised nor themes accepted without team consensus. This process, alongside the inductive analytic approach (*Section 2.7*), provided checks against confirmatory bias. AS’s religious identity may have facilitated rapport but risked over-assuming shared perspectives; this was mitigated by focusing on health rather than theology, and by team discussions testing interpretations against diverse views.

The only religious tradition the researcher had not personally observed in physical worship was Judaism. He was equally relaxed and comfortable interviewing all Faith-leaders, observing them to be equally relaxed, interested, engaged, and fascinating. The interviewer (AS) is male. This identity may have influenced participants’ responses, particularly given the predominantly male leadership of the faith institutions approached. However, his clinical background and the study’s focus on health rather than religious or spiritual matters may have mitigated potential gender-related biases.

Member checking was not undertaken as the analysis focused on identifying patterns across the sample rather than verifying individual accounts. Returning transcripts could have introduced retrospective rationalisation, obscuring initial narratives (41). Instead, rigour was supported through triangulation strategies described in *Section 2.9*: investigator (team coding review), methodological (interviews with field notes and reflexivity journals), and theoretical (inductive analysis complemented by Weberian interpretation).

### 2.9 Triangulation

Triangulation strategies were employed: investigator triangulation through review of the coding process by multiple team members (AS, HE, SG); data triangulation through purposive sampling of leaders from diverse religious traditions; methodological triangulation by combining interview data with field notes and reflexivity journals; and theoretical triangulation by complementing inductive thematic analysis with Weber’s theory of religious authority to interpret patterns of institutional legitimacy and partnership conditions.

### 2.10 Ethics approval and consent to participate

Ethics approvals were obtained from The University of York Health Sciences Research Governance Committee, and The Institutional Review Board of The Nigerian Institute of Medical Research. All participants provided informed consent. Trained researchers explained the information leaflet to each individual and addressed any questions. Both verbal and written consent were obtained; verbal consent was confirmed and documented via electronic signature from each participant. Electronic signatures were obtained via a secure online consent form. Signed consent forms are stored on a password-protected, encrypted university server, accessible only to the research team. The study was conducted during the COVID-19 pandemic, which necessitated remote interviews and may have affected recruitment and responses. No minors (individuals under 18 years) were enrolled, as the study was restricted to adults. The study followed the Declaration of Helsinki. Consent for publication is not applicable, as this manuscript contains no individual person’s identifiable data in any form.

The completed COREQ checklist and the codebook are provided as Supplementary Files 1, 2, respectively.

## 3 Results

### 3.1 Informants’ characteristics

Table 2 shows characteristics of faith leaders and their institutions, parishes, or congregational units. The faith-leaders were one female and seven males. Four were aged 30–50 and four aged 50–70. Each conveyed personal conviction that they occupied their roles by somewhat divine assignment to impact others for good, expressing closeness to those they served and emotional investment in their communities. Each was the main faith leader at their local faith institution. Three congregations were small (local permanent membership below 100); four were medium (membership under 1,000); one was large (membership over 1,000). All localised their influence within their local communities and across Lagos state. Three declared regional influence (across at least one other state in Southwest Nigeria beyond Lagos). Three declared some form of national influence (past or current countrywide leadership role, work, or position). Three declared that previous social, charitable, or other activity by their parishes had extended nationally in impact. The leaders’ health literacy may reflect their relatively educated backgrounds (as indicated in Table 2) and may not be representative of all faith leaders in Lagos.

### 3.2 Thematic analysis

Table 3 presents an overview of the four analytical and 16 descriptive sub-themes generated.

**Table 3:** Themes emerging from the data analysis.

Analytical themes	Brief meaning	Sub-themes
Deficient health system	The Health System is rendered deficient to address hypertension health by longstanding infrastructure and socioeconomic challenges	The current state Fundamental shortcomings Reality
Severely impacted society	Society bears a high impact from hypertension due to lack of collective effort and innovation	Defective understanding Hierarchy of needs Severe social impact Unavoidable responsibility Potential benefits
Underutilised faith institutions	Faith institutions are insufficiently used in hypertension mitigation despite presenting opportunities for collaboration	Unrewarding effort Potential for optimism and collaboration Capable assets for innovation Advocates for multi-institution collaboration
Possible change of approach	A change in approach to hypertension mitigation leveraging innovation, community engagement and local resources is possible.	Advocates for change Non-governmental solutions are possible Resources and leadership drive change Solutions are possible through faith institution influence

### 3.3 Theme one: deficient healthcare system

This theme captures faith-leaders’ perspective that the health system is rendered deficient for hypertension care by longstanding infrastructure and socioeconomic challenges. They all believed the healthcare system lacks adequate resources to support healthy behaviour, early detection, and comprehensive hypertension management. They thought significant disparities in healthcare quality, quantity, distribution, and access compel out-of-pocket payments for private care. Hence, early hypertension detection and comprehensive management may not be as widespread as needed, given already poor hypertension awareness and adverse economic conditions. These challenges create a mostly negative reality for faith institution users regarding hypertension diagnosis and management, characterised by uncertainty, fear, and anxiety. When reflecting on health system inadequacies, all Faith-leaders were carefully conciliatory, non-critical, and viewed the government’s role as irreplaceable. They all thought private healthcare was more accessible than the public system but unaffordable for most. This theme was consistent across all Faith-leaders and comprised three descriptive sub-themes: The current state, Fundamental shortcomings, and Reality.

#### 3.3.1 The current state

Faith-leaders believed that inadequate health system infrastructure, inefficient healthcare delivery, and a culture preoccupied with socioeconomic issues (rather than health) have contributed to insufficient hypertension knowledge and awareness, and prevented hypertension from being perceived as requiring deliberate protection or improvement. They reflected that the healthcare system is burdened by questionable governance, outdated infrastructure, and inadequate resources, rendering it incapable of modernisation or effectively addressing the worsening hypertension epidemic. Their collective view is that the healthcare system cannot facilitate healthy behaviour change, offer routine free health checks, or adequately train, deploy, and fairly compensate sufficient healthcare staff.

*“The healthcare situation is shambolic. Death because of hypertension continues because of poor awareness and poor belief.” (Respondent 3, Middle Aged Male ATR priest).*

#### 3.3.2 Fundamental shortcomings

Faith-leaders identified several fundamental obstacles hindering healthcare provision and hypertension management: inadequate facilities, insufficient infrastructure maintenance, inadequate therapeutics, and the absence of a functional primary healthcare system or alternative disease surveillance. These factors create a discouraging environment for already overstretched healthcare workers and exacerbate health outcome inequalities by forcing individuals to manage their health with whatever resources they have, rather than relying on the healthcare system.

*“The citizens do not seem to have that confidence in a government that their future is secure.” (Respondent 2, Middle Aged Male Pentecostal Christianity Pastor).*

*“The future is actually very bleak because things might just become too terrible. Hypertension might cause a lot of hopelessness in the society. If nothing is done about it the workforce will be affected, the economy that is already not good will continue to be depreciating.” (Respondent 8, Male Catholic Christianity Priest under 50 years old).*

#### 3.3.3 Reality

All Faith-leaders believed that widespread unemployment, inadequate health promotion, and nearly non-existent health surveillance are part of the reality, with insufficient government action and lack of confidence in the government’s stewardship of healthcare. People are preoccupied with combating poverty and surviving economic hardship, relegating health education to the background. They perceived a prevailing pessimism surrounding the prospects of enhanced healthcare and hypertension management for the most marginalised in society, including faith institution users. This pessimism could worsen without changes in health system governance or improvement in current socioeconomic realities.

*“The government is responsible for the population of the country. Poverty needs to be dealt with generally. Health checks need to be made free. Increase awareness for the citizen. The level of poverty is there. We do not have the means. Make everything free. I’m talking of healthcare. If they made it free, people will have access to it. If someone is not down they will not attend the hospital.” (Respondent 1, Middle aged male Sunni Imam).*

### 3.4 Theme two: severely impacted society

This theme captures society’s high hypertension burden due to lack of collective effort and innovation. Faith-leaders believed that absent collaboratively designed or widely implemented health education programmes promoting beneficial hypertension behaviours may worsen hypertension’s existing societal burden. Responsibility for hypertension awareness should not rest solely on government but be shared among the healthcare system, non-governmental organisations, faith institutions, and society members. Longstanding infrastructure and capability challenges within the healthcare system require innovative thinking to facilitate burden sharing. All Faith-leaders agreed that managing hypertension in Lagos is highly stressful and challenging. They unanimously believed that congregations often confuse hypertension with psychological stress (rather than being entirely ignorant), and that poverty and economic stresses significantly influence congregation members’ health-related decisions, often causing suffering due to inadequate public healthcare. This theme was consistent across all Faith-leaders and comprised five descriptive sub-themes: Defective understanding, Hierarchy of needs, Severe social impact, Unavoidable responsibility, and Potential benefits.

#### 3.4.1 Defective understanding

In Lagos’ context of heavy societal hypertension burden and health system inadequacies, accurate understanding of health and disease is particularly relevant to influencing health-related behaviours. Faith-leaders thought that individual and collective ignorance about hypertension, poor understanding, and superstitious beliefs have combined to make hypertension a disease usually diagnosed late. All participating faith-leaders described this theme.

*“When something is wrong with Africans they will think it is a spiritual attack, somebody is hypnotising him or her. Hypertension is a bodily problem. A physical problem, not a spiritual problem.” (Respondent 3, Middle Aged Male ATR priest).*

*“Many people just do not really understand… People tend to find out only when they attend hospital.” (Respondent 6, Female ATR Priestess under 50 years old).*

#### 3.4.2 Hierarchy of needs

Faith-leaders believed that high poverty levels compel congregation members to prioritise needs hierarchically, often resulting in inappropriate health-related and healthcare decisions. A typical example: prioritising daily needs over healthcare expenditure, risking less favourable long-term health outcomes.

*“When you see somebody who is not able to feed his or herself or the family, you are now telling the person to go and pay money for healthcare. So the person will just inform you that the first thing is meal. They do not care until maybe the illness gets to a level where it is very severe. Poor availability, high cost and poverty is forcing people to make decisions that are not the best for them. That’s how the situation is. It’s the system we have been moving with. That’s why I said initially, it is the mercy of God that keeps the people alive.” (Respondent 1, Middle aged male Sunni Imam).*

#### 3.4.3 Severe social impact

Faith-leaders recognised that suboptimal decisions in Lagos, amidst its high hypertension burden, frequently lead to preventable morbidity and premature mortality. They believed that congregations of all faiths, like other society members, suddenly fall victim to hypertension during their prime. Leaders from Islam, Christianity, and African Traditional Religion noted fatalities affecting diverse individuals: young, vibrant, educated, intelligent, productive, aspirational, impoverished, affluent, parents, and even Faith-leaders. They believed that premature deaths and preventable disabilities from hypertension within their congregations remain largely hidden, yet impose substantial economic burdens on affected families and indirectly affect wider society.

*“Less than two weeks ago, one of us…. died of hypertension.” (Respondent 5, Male Ahmadi Islam Imam under 50 years old).*

*“We lost someone in my former church parish. He was more or less like the leader of the men. A very up and doing person… the experience of another young valuable person within my institution who unfortunately died suddenly from a stroke linked to hypertension.” (Respondent 4, Middle Aged Male Pentecostal Christianity Pastor).*

*“Hypertension has a high impact on the society by decimating the human resources and cutting lives short. It impacts the society negatively in terms of them not being available to contribute to the society because they have health challenges due to blood pressure.” (Respondent 5, Male Ahmadi Islam Imam under 50 years old).*

#### 3.4.4 Unavoidable responsibility

Faith-leaders believed that raising hypertension awareness is an inevitable collective responsibility, shared among the general public, clergy, faith institutions, non-governmental organisations, and government. They reflected on the potential benefits of government-supported hypertension awareness within faith institutions through health system resource allocation.

*“The government has responsibilities but individuals also have responsibilities. Everybody in the society has responsibilities including NGOs as well as religious and Faith-leaders like myself.” (Respondent 3, Middle Aged Male ATR priest).*

#### 3.4.5 Potential benefits

Faith-leaders believed that collaborative efforts and shared responsibility are advantageous and can take various forms: integrating government poverty alleviation into hypertension awareness campaigns, leveraging religious scripture for health education, and directly engaging local communities through mass media (e.g., radio broadcasts). Hypertension’s persistent, significant societal impact calls for innovative solutions. Innovation among non-governmental organisations and faith institutions collectively addressing hypertension awareness could spread potentially lifesaving interventions throughout Lagos society.

*“For example using the media, like radio programmes. A large number of people can be reached, even elderly ones. Radio reaches a large population. It is a good means or tool of awareness.” (Respondent 6, Female ATR Priestess under 50 years old).*

### 3.5 Theme three: underutilised faith institutions

This theme captures Faith-leaders’ view that faith institutions are insufficiently used in hypertension mitigation despite opportunities for collaboration. Faith-leaders expressed that faith institutions adhere to religious scripture guidance on maintaining pure and healthy lifestyles, and regard advocating for good health (including addressing poor hypertension awareness) as part of their responsibilities. They believed that large segments of society feel socially comfortable in religious worship environments due to the deep influence of religious philosophies. Despite consistent challenges with limited resources and implementation logistics, this environment has been exposed to viable solutions for addressing hypertension. However, opportunities for grassroots collaboration are being missed because faith institutions are not deploying their resources to complement existing health system or NGO programmes. Nevertheless, all Faith-leaders viewed faith institutions’ pragmatic self-help mitigation activities as humanitarian and expressed willingness to collaborate given the right conditions. This theme comprised four descriptive sub-themes consistently represented among all Faith-leaders: Unrewarding effort, Potential for optimism and collaboration, Capable assets for innovation, and Advocates for multi-institution collaboration.

#### 3.5.1 Unrewarding effort

Faith-leaders across all religions viewed alleviating human suffering as a fundamental moral duty of faith institutions. Consequently, they allocate resources to support general health awareness, periodically organising health events that leverage donated services from medical professional members to flexibly cater to users’ healthcare needs. However, they expressed frustration about the limitations of hypertension mitigation achievable through their health ministries and discontent with the perceived lack of innovative solutions to address preventable suffering caused by hypertension, attributing this to inadequate healthcare provision.

*“Most prefer to just hear the Dawah, the lecture and after that move out. Whereas the healthcare, they are outside the mosque waiting for people to come outside. There are only few that attend. We cannot compel individuals to attend. But those who know the importance of these health checks do attend these tests.” (Respondent 1, Middle aged male Sunni Imam).*

#### 3.5.2 Potential for optimism and collaboration

Faith-leaders viewed potential hypertension solutions with a mixture of optimism and resignation. They found individual faith institutions’ health ministry efforts encouraging but considered the absence of collaborations between them counterintuitive. They reflected on the lack of collaborations, coordination, and cooperation among multiple faith institutions’ health ministries, suggesting this may stem from confusion about their potential impact. Despite societal healthcare failures, many people enjoy reasonably good health, attributed to fortune or ‘Divine grace.’ Nevertheless, all remain optimistic that viable hypertension solutions could be found. The Ahmadi Islamic imam and the Catholic priest emphasised direct charitable activities to benefit the poor and common person.

*“Let us start working toward this direction and let us see how the country at large can be set free from the hypertension, high blood pressure and the rest of it.” (Respondent 7, Male Judaism Rabbi under 50 years old).*

*“This goal of community and church working together, the church being one, religious institutions working toward a common goal, the church spreading love, can be realised. It can be realised if different churches can work together. Health is expensive. A lot of persons in Nigeria are finding it difficult to access health facilities. I think through team-work, NGO collaboration with churches and mosques can team up.” (Respondent 2, Middle Aged Male Pentecostal Christianity Pastor).*

#### 3.5.3 Capable assets for innovation

Faith-leaders believed that faith institutions are crucial assets for addressing hypertension health, given their multifaceted capabilities: intimate grassroots community connections and the ability to tailor health and education campaigns to religious faith. They thought faith institutions are enthusiastic about collaborating with governmental, non-governmental, corporate, and charitable bodies, and are open to implementing innovative health programmes and partnerships to fulfil their public service obligations, including addressing hypertension health.

*“If there are people that want to partner with the church to make it better, definitely I am sure that would be welcome. Whatever is good for the people, I am sure the church would be ready to be a part of it. The church is a care organisation: whatever the church can do to help people manage their blood pressure, we’ll be favourably disposed. The church is not just interested in people going to heaven. The church is also interested in people living a healthy life.” (Respondent 4, Middle Aged Male Pentecostal Christianity Pastor).*

#### 3.5.4 Advocates for multi-institution collaboration

Highlighting faith institutions’ limited resources, Faith-leaders underscored the importance of government accountability in providing essential resources and utilities for a cohesive, functioning society. They believed the government is uniquely placed to harness society’s diverse capabilities and respond effectively to initiatives and opportunities, and that they, their institutions, and broader society naturally gravitate toward following government leadership in health initiatives, particularly those capable of attracting private or non-governmental organisations. However, to facilitate hypertension initiatives within faith institutions, the government must accept assistance from non-governmental organisations, corporate bodies, and faith institutions, and actively promote collaboration between these entities and the healthcare system.

*“The best way … is for the government to prioritise hypertension, the religious organisations to prioritise it. Whatever the government takes as priority the citizens also take as priority. We’ve given support to the state government.” (Respondent 5, Male Ahmadi Islam Imam under 50 years old).*

### 3.6 Theme four: possible change of approach

This theme captures respondents’ belief that a change in approach to hypertension mitigation — leveraging innovation, community engagement, and local resources — is possible. Faith-leaders believed that resources within faith institutions and local communities offer ample opportunities to guide this change. Prioritising faith institutions’ identity as compassionate organisations, along with their self-assumed moral obligation to serve congregations and wider society, could expedite harnessing their resources for hypertension health. They understand hypertension as preventable and treatable, and are open to innovative use of their ideas and networks. They are willing to commit faith institution resources to integrate governmental and non-governmental initiatives into existing health ministries, demonstrating readiness to embrace collaborations. All Faith-leaders identified nutrition practices as a potentially crucial focus for hypertension interventions within diverse faith communities. A Pentecostal Christian pastor and the Jewish rabbi particularly emphasised healthy nutrition as a key intervention component. The African Traditional Religion priest, Sunni Islamic imam, and Jewish rabbi all emphasised proactive engagement with local and state authorities to advocate for healthcare in their respective faith communities. This theme consistently emerged across all Faith-leaders and comprised four descriptive sub-themes: Advocates for change, Non-governmental solutions are possible, Resources and leadership drive change, and Solutions are possible through faith institution influence.

#### 3.6.1 Advocates for change

Faith-leaders believed that a desirable change in approach would require greater transparency in resource allocation and heightened responsiveness to society’s evolving health needs. Enhancing healthcare workforce motivation, providing further training, and ensuring fair remuneration could have significant impact. African Traditional Religion leaders believed that training and deploying traditional and herbal medicine practitioners in healthcare roles could improve accessibility.

*“We wrote to the state commissioner for youth and development. We wrote to the commissioner for wealth creation. We wrote to the commissioner for Local Government and community affairs. We have commissioner for environment and water resources. So we believe that all these ministries they interphase with youth one way or the other. The reason why we plan to meet them is to say that we are here. We’ve given support to the state government in terms of having a greater Lagos.” (Respondent 5, Male Ahmadi Islam Imam under 50 years old).*

*“If the government policy focuses on majorly on the healthcare and they try as much as possible to provide the means whereby the citizens will have access – the majority of the populace have access to these healthcare by bringing in free. This is very very important.” (Respondent 1, Middle aged male Sunni Imam).*

#### 3.6.2 Non-governmental solutions are possible

Faith-leaders emphasised acknowledging resource constraints within the public healthcare system, noting that exclusive reliance on it to address hypertension is unrealistic. They believed hypertension prevention and management could extend beyond the healthcare system to include faith settings and local communities. With support from governmental and non-governmental organisations, faith institutions could innovate and lead hypertension prevention by mobilising resources from both faith institutions and local communities. Christian, Muslim, and African Traditional leaders believed such initiatives could involve collaborations across multiple congregations and faiths.

*“… if the churches and mosques in collaboration with NGOs can team up together to see how they can provide healthcare to subsidise what the government is doing, because the government cannot do everything alone.” (Respondent 2, Middle Aged Male Pentecostal Christianity Pastor).*

#### 3.6.3 Resources and leadership drive change

Faith-leaders believed that the most effective hypertension healthcare is delivered at grassroots level, utilising locally generated knowledge and solutions to address community-specific challenges. The resources or leadership necessary to achieve such goals have yet to be attained. They found it particularly frustrating that the financial resources needed for healthcare infrastructure and supporting healthcare professionals exceed the means of both faith institutions and local communities. Hence, a change in government approach is essential for progress in hypertension health.

*“Getting healthcare at the grassroots level so that there will not be the need to travel far to get healthcare. Because healthcare is a very important issue. For many people having to travel too far has led to people losing their lives before they get the medical help needed. Frequently it is a dead man that is attended to. That is part of a lot of the issues.” (Respondent 7, Male Judaism Rabbi under 50 years old).*

*“But what of the funding? Because if I tell you okay this is what and what will be done for us to get this thing done. I may decide to write to the government and tell government what to be done. They will ask you there is no money to push this case. That’s what they will tell you.” (Respondent 7, Male Judaism Rabbi under 50 years old).*

#### 3.6.4 Solutions are possible through faith institution influence

Faith-leaders explained that through their health ministries, faith institutions already use the religious worship environment to expose users to informal health promotion and counselling, sometimes enlisting charitable or secular organisations to deliver these activities. There may be opportunities for innovative hypertension health strategies integrated into existing faith institution health ministries. Elements from religious culture, doctrines on charity, lifestyle, dietary laws, and existing faith community practices could be adapted for hypertension interventions.

*“Because in Nigeria most of the population believe in God, Mosques are full. Churches are also full up. So these two institutions have very large populations on the day of worship. Government can target these areas and move down where the religious institutions are located. If they target churches and all kinds of faith institutions, because Nigerians are fond of these two religions… I’m sure most of the population will know their health status.” (Respondent 1, Middle aged male Sunni Imam).*

### 3.7 Unexpected findings

Beyond the four main themes, three unexpected observations emerged from the data.

First, faith leaders’ critiques extended beyond the health system to include past partners—non-governmental organisations and academic researchers. They described being approached for data collection or short-term health campaigns, only to be abandoned once the project concluded.

Second, faith leaders demonstrated strategic pragmatism that complicated any simple narrative of religious altruism. They expressed willingness to collaborate, but did so conditionally, articulating specific and negotiable prerequisites for partnership.

Third, the emotional toll of hypertension on faith leaders themselves was striking and unanticipated. They described congregants’ suffering and their own distress at witnessing preventable deaths, particularly among young, productive members, and spoke of the suffering they observed within their communities.

## 4 Discussion

This qualitative study explored eight Lagos, Nigeria faith leaders’ perspectives on health, hypertension, and their institutions’ potential roles in hypertension prevention and management. Four interconnected themes emerged: a deficient health system, severely impacted society, underutilised faith institutions, and a possible change of approach. This discussion advances a single overarching argument: faith leaders occupy a paradoxical position – sophisticated diagnosticians of health system failure and strategic thinkers about partnership conditions, yet structurally excluded from formal hypertension strategies. This paradox stems from historical patterns of extractive collaboration, an epistemological hierarchy privileging clinical over community knowledge, and the absence of a theoretical framework for understanding religious authority as a health asset. Resolving this paradox may require moving beyond tokenistic engagement to genuine, reciprocal, and theoretically informed partnerships. This is a proposition that could be tested in future research.

### 4.1 Theoretical framing

Max Weber’s theory of religious authority provides a powerful analytical lens (39). Weber distinguished three ideal types of legitimate authority: traditional (derived from institutional lineage and scripture), rational-legal (derived from formal position within hierarchies), and charismatic (derived from personal qualities and perceived divine calling). Lagos faith leaders derive influence from all three simultaneously. This compound authority explains why their conditional willingness to collaborate is so consequential. Unlike secular community health workers whose authority is delegated and revocable, Faith-leaders’ authority is ontologically grounded in religious belief systems that predate and transcend any particular health intervention. When they endorse or withhold endorsement from a hypertension programme, they activate deep trust within their congregations – a dynamic largely underexplored in faith and health literature, which has treated Faith-leaders as convenient access points rather than authority figures whose legitimacy shapes intervention uptake (12, 15).

Weberian theory also illuminates Faith-leaders’ critiques of past partnerships. They described collaborations as extractive – partners sought access to congregations for data collection or short-term campaigns without sustained support. From a Weberian perspective, this is not merely poor practice but a fundamental misunderstanding of religious authority. Faith-leaders risk their legitimacy when facilitating interventions that do not benefit their congregations. Extractive partnerships therefore deplete the authority that makes Faith-leaders effective partners. Faith-leaders’ insistence on reciprocity, sustainability, and tailoring is not strategic pragmatism alone but a rational defence of institutional legitimacy. This finding challenges the implicit model in much global health literature, which assumes community leaders will cooperate with externally designed interventions if consulted (42). This study’s data suggest consultation is insufficient; Faith-leaders require demonstrable, sustained benefit to their congregations as a condition of continued collaboration. Their conditional willingness to collaborate is not reluctance but a rational defence of institutional legitimacy, which can be understood through Weber’s framework of religious authority, helping make sense of how faith leaders view their institutional legitimacy (39). While Weber’s framework provides a productive analytical lens, it is one of several theoretical perspectives that could inform understanding of faith-health partnerships.

### 4.2 Comparative analysis

The Lagos findings can be usefully compared with faith-based health research in other settings. In Ghana, studies document how religious beliefs shape hypertension understanding, with some patients interpreting hypertension as a spiritual challenge requiring both medical and faith-based responses (30). This parallels the Lagos data, where Faith-leaders reported congregants often confuse hypertension with psychological stress or spiritual attack. However, the Ghanaian literature focuses on individual patient beliefs, whereas the Lagos study examined institutional perspectives – a significant difference in analysis level. In South Africa, church-based healthy lifestyle programmes have shown feasibility and acceptability, but sustainability remains challenging (43). This mirrors the Lagos finding that faith institutions’ health ministries are often volunteer-run and intermittent. The South African literature has developed practical implementation models, whereas the Lagos study contributes deeper theoretical understanding of why sustainability challenges persist. In Kenya, faith-based organisations have contributed to primary healthcare delivery, but government–faith partnerships remain *ad hoc* and under-resourced (44), highly consistent with the Lagos finding that faith institutions are underutilised despite demonstrable willingness. The Kenyan experience suggests policy recognition alone is insufficient without dedicated resources and accountability mechanisms.

Comparison with the African American church literature in the United States is particularly instructive. African American churches have been extensively studied as sites for hypertension and cardiovascular interventions, with evidence of short-term blood pressure reductions and improved health behaviours (43, 45). However, that literature operates within a different health system context – one with established clinical infrastructure, insurance coverage, and professionalised faith-based intervention models. Lagos Faith-leaders operate where the formal health system is often inaccessible or unaffordable, making faith institutions not merely adjuncts but potential substitutes for certain primary care functions (screening, basic health education, referral coordination). This difference has important implications: interventions developed in high-income settings cannot be simply adapted; they require fundamental rethinking for contexts where faith institutions may be the most accessible, trusted health asset available.

Across these diverse settings, several similarity patterns emerge. Faith-leaders consistently report similar assets – trust, reach, moral authority, and grassroots connections – and similar constraints – limited resources, volunteer dependence, and intermittent health ministry activity. These consistencies suggest that faith institutions share core features transcending national and religious boundaries. However, the Lagos findings are distinct in one important respect: Faith-leaders articulated a more structurally critical analysis of health system failure than typically reported. They did not attribute poor hypertension outcomes to individual ignorance or sin but to inadequate infrastructure, chronic underfunding, governance failures, and poverty. This structural framing aligns with social determinants of health literature but is relatively rare in faith and health research, which has focused on individual behaviour change rather than systemic critique (12, 15). The distinctiveness of the Lagos findings may reflect Nigeria’s particularly severe health system challenges, including one of the lowest per capita health expenditures globally and a federal governance structure that has fragmented primary healthcare delivery (3, 5–7).

### 4.3 Unexpected findings

Three unexpected findings warrant explicit discussion.

First, the critique of past partners—including non-governmental organisations and academic researchers—is rarely reported in the faith and health literature, which tends to present partnerships as unproblematic or focus on barriers within faith institutions rather than external partners (46–50). Researchers and practitioners must scrutinise their own practices, not just Faith-leaders’ readiness (46, 47).

Second, the strategic pragmatism exhibited by faith leaders challenges the romanticised assumption—pervasive in practice yet largely unexamined in the literature—that faith institutions are motivated solely by religious duty. Faith-leaders are moral actors, but also institutional leaders responsible for their congregations’ welfare and their organisations’ sustainability. Their pragmatism is not cynicism; it is rational stewardship.

Third, the striking and unanticipated emotional toll of hypertension on faith leaders themselves has significant implications. This emotional dimension, while documented in some empirical studies (51), is rarely reflected in policy frameworks or standard quantitative surveillance, yet it remains central to understanding Faith-leaders’ urgency and willingness to act. It also suggests that Faith-leaders may risk compassion fatigue or burnout if interventions do not include support for their own wellbeing (51).

### 4.4 Implications for policy, practice and research

#### 4.4.1 Implications for policy

Lagos and Nigeria policymakers should recognise faith institutions not as peripheral or optional partners but as existing, functioning health assets. The National Health Promotion Policy already names faith institutions as settings for health promotion, but this has not translated into resource allocation or systematic engagement (52). Three concrete policy actions are recommended: include faith institution health ministries in routine health system mapping by Local Government Area health departments; establish formal, funded liaison roles between primary healthcare facilities and faith networks to facilitate referral pathways and shared accountability; and allocate dedicated resources for capacity building of faith institution health ministries, including training in blood pressure measurement, basic hypertension management, and referral protocols. These actions could be piloted in selected Local Government Areas, with evaluation focused on feasibility, acceptability, and early outcomes before wider scale-up.

#### 4.4.2 Implications for practice

Health practitioners and non-governmental organisations seeking partnerships with faith institutions should adopt four principles derived from this study. Reciprocity: partnerships must benefit faith institutions, not extract congregants. This could include training, equipment, small grants, or recognition. Sustainability: volunteer-led models have limits. Partners should explore sustainable resourcing models, including linking faith health ministries to existing government or insurance-based financing mechanisms. Tailoring: interventions must adapt to congregational characteristics: religious doctrine, socioeconomic composition, age profile, and existing health activities. Moderate tailoring (e.g., adapting health messages to religious framing, scheduling screening around worship times) is feasible and effective (53). Trust-building: past negative experiences mean initial collaborations should be small, transparent, and delivered as promised. Partners should invest time in relationship building before expecting Faith-leaders to mobilise congregations.

Potential harms of faith-based hypertension interventions include over-medicalisation, stigmatisation, and perceptions of exclusion—including self-exclusion where faith institutions’ cultural or religious environment feels unwelcoming. Mitigation could involve thoughtful integration of health messages within worship practices, avoiding public identification of those with hypertension, and ensuring accessibility. Co-production with diverse stakeholders (congregants, community members, health workers) and consultation with non-attendees can help identify barriers and ensure inclusivity.

#### 4.4.3 Implications for research

Future research should address six gaps identified by this study: evaluate the effectiveness of faith institution-placed hypertension screening and referral pathways in Lagos (ideally through pragmatic trials or quasi-experimental designs); explore congregants’ perspectives (including non-regular attendees) to complement faith leader accounts; investigate potential harms of faith-based interventions (exclusion of non-congregants, reinforcement of health-related stigma, over-spiritualisation of medical conditions); conduct comparative studies across Nigerian regions (e.g., predominantly Muslim north, oil-rich south) to test transferability; use implementation science frameworks to understand what facilitates or hinders sustained health system–faith institution partnerships; and apply Weberian and other theoretical frameworks to faith and health research, which has been notably under-theorised despite its empirical richness. Future research could also triangulate leaders’ accounts by incorporating document review, secondary health statistics, or data from congregants and health workers to corroborate or expand on the perspectives captured in this study.

### 4.5 Strengths and limitations

This study has several strengths. It is the first qualitative study, to our knowledge, specifically examining Lagos faith leaders’ perspectives on hypertension interventions, addressing a significant evidence gap. Purposive sampling achieved exceptional diversity across religious traditions, including underrepresented groups (African Traditional Religion, Judaism, Ahmadiyya Islam). Inductive thematic analysis remained grounded in participants’ own words, preserving narrative richness and authenticity. Weberian theory provides a novel analytical lens advancing theoretical understanding of faith-based health partnerships. The inclusion of a female faith leader (African Traditional Religion priestess) adds valuable gender diversity often absent in faith-leadership research.

Limitations must be acknowledged. The study was conducted during the Covid-19 pandemic, necessitating remote interviews, which may have affected recruitment and interpersonal engagement depth. Social desirability bias may have influenced faith leaders to present themselves and their institutions as willing partners. Remote interviews may have limited rapport and observing nonverbal cues. These factors were partially mitigated by using video where possible and collecting field notes and reflexivity journals to capture contextual observations. Despite extensive recruitment efforts, leaders from Buddhism and Hinduism could not be included, though their absence is unlikely to substantially affect findings given the demographic dominance of Islam and Christianity in Lagos. The absence of a white garment Christian church leader is a limitation, though their perspectives may align with those of other Christian leaders interviewed. Tribal and ethno-linguistic identities were not used as analytical variables, which may have obscured intra-faith variations, though the diversity of ethnic backgrounds within Lagos religious faiths likely mitigates this concern. The sample size (*n* = 8) was appropriate for the exploratory, in-depth aims of this study, but findings are context-specific to Lagos and may not be directly transferable to other regions without careful adaptation to local health system contexts, religious demographics, and governance structures. The Weberian theoretical lens, while productive, is one of several possible frameworks; alternative lenses (e.g., structural vulnerability, social capital, diffusion of innovations) might illuminate different data aspects. Finally, as with all qualitative research, findings represent Faith-leaders’ accounts, not direct measures. Where Faith-leaders reported poor hypertension knowledge or preventable deaths, these are their perceptions, which may not perfectly correspond to clinical or epidemiological data, but are nonetheless important because they shape their priorities and actions.

The sample was predominantly male (one female, seven males), reflecting the leadership demographics of the institutions that participated, which limits generalisability to female-led faith institutions. Differences in congregational size and theological orientation were also present. While this study did not examine how these factors influence willingness or capacity for interventions, future research could explore these dynamics. The study was designed to capture leadership perspectives only; congregant or frontline health worker views were outside its scope. However, they may be more accurately transferable to contexts that are sufficiently similar to Lagos.

## 5 Conclusion

Addressing the four research questions in turn, this study found that faith leaders view congregants’ hypertension experiences as entangled with poverty, psychological stress, and spiritual beliefs; perceive societal hypertension awareness as severely deficient despite high prevalence; identify healthcare availability as fundamentally constrained by inadequate infrastructure, chronic underfunding, and unaffordability; and regard their institutions as willing but underutilised partners capable of hosting tailored interventions, provided collaborations are reciprocal and respectful of their institutional legitimacy. The core contribution: Lagos faith leaders are not passive recipients of externally designed interventions but sophisticated diagnosticians of health system failure and strategic thinkers about partnership conditions.

The underutilisation paradox—where willing, capable faith institutions are excluded from formal hypertension strategies—may require moving beyond tokenistic engagement to genuine, reciprocal, theoretically informed partnerships that respect their leaders as equal partners, not convenient access points. This proposition requires further empirical testing. Without such a shift, a valuable asset will remain underused, and preventable hypertension deaths will persist. With such a shift, Lagos faith institutions could become a model for faith-based hypertension interventions across Sub-Saharan Africa – not by replicating high-income country templates, but by developing contextually tailored, sustainably resourced, and theoretically grounded approaches that leverage religious leaders’ unique authority as trusted guardians of community health.

## Data Availability

The raw data supporting the conclusions of this article will be made available by the authors, without undue reservation.
