# Supplementary material for: The perspectives of Lagos faith-institution leaders on health, hypertension, and faith-based hypertension interventions: a qualitative interview study
Source: Front Public Health. 2026 Sep 9;14:1925205. doi: 10.3389/fpubh.2026.1925205 (PMC13597788; doi:10.3389/fpubh.2026.1925205)
Supplement: Supplementary file 1 [file Data_Sheet_1.pdf]

COREQ (COnsolidated criteria for REporting Qualitative research) Checklist

Study Title: The perspectives of Lagos faith-institution leaders on health, hypertension, and faith-based hypertension interventions: a qualitative interview study

Item	Guide Questions/Description	Answer	Reported on Page number
**Domain 1: Research team and reflexivity**			
Personal Characteristics			
1. Interviewer/facilitator	Which author/s conducted the interview or focus group?	AS conducted the interviews	Page 4
2. Credentials	What were the researcher's credentials? E.g. PhD, MD	AS: MD, MSc, MPH, PhD. HE: PhD. SG: PhD. OT: PhD.	Page 4
3. Occupation	What was their occupation at the time of the study?	AS: Doctoral researcher; HE, SG, OT: Academic staff, University of York	Page 4
4. Gender	Was the researcher male or female?	AS (male), HE (female), SG (female), OT (female). Interviewer gender discussed in reflexivity.	Page 4 – 6
5. Experiencing and training	What experience or training did the researcher have?	AS formally trained in qualitative research methods, with prior experience conducting qualitative interviews and worked as a medical doctor internationally. HE, SG and OT were also trained and were more experienced to supervise AS.	Page 4
Relationship with participants			
6. Relationship established	Was a relationship established prior to study commencement?	No prior relationship existed between the researchers and any participant.	Page 4
7. Participant knowledge of the interviewer	What did the participants know about the researcher? e.g. personal goals, reasons for doing the research	Participants were informed that AS was a doctoral researcher at the University of York and that the study was part of his doctoral research.	Page 4
8.Interviewer characteristics	What characteristics were reported about the interviewer/facilitator? e.g. Bias, assumptions,	AS's clinical background and interest in hypertension were shared during the informed	Page 4, 6

	reasons and interests in the research topic	consent process. Reflexivity section discusses positionality.	
**Doman 2: study design**			
Theoretical framework			
9. Methodological orientation and Theory	What methodological orientation was stated to underpin the study? e.g. grounded theory, discourse analysis, ethnography, phenomenology, content analysis	The methodological orientation was thematic analysis, pragmatically chosen as the most suitable approach for the research objectives.	Page 4, 6
Participant selection			
10. Sampling	How were participants selected? e.g. purposive, convenience, consecutive, snowball	Purposive sampling; maximum variation across religious traditions.	Page 2
11. Method of approach	How were participants approached? e.g. face-to-face, telephone, mail, email	Phone calls, Internet and social media searches; invitations to over 91 institutions.	Page 2
12.Sample size	How many participants were in the study?	Eight faith institution leaders.	Page 2
13. Non-participation	How many people refused to participate or dropped out? Reasons?	From 91 faith institutions contacted, 8 faith leaders participated. Non-participation due to late-stage withdrawals, internal bureaucracy, logistical challenges, or unexplained reasons.	Page 2 – 3
Setting			
14. Setting of data collection	Where was the data collected? e.g. home, clinic, workplace	Remote (Zoom, telephone, WhatsApp).	Page 3
15. Presence of non-participants	Was anyone else present besides the participants and researchers?	No other individuals were present during interviews.	Page 4
16. Description of sample	What are the important characteristics of the sample? *e.g. demographic data, date*	Diverse religious traditions; one female, seven males; age range 30–70 years. Please see Table 2.	Page 2, 5
Data collection			
17.Interview guide	Were questions, prompts, guides provided by the authors? Was it pilot tested?	Yes – Please see Table 1.	Page 3

18. Repeat interviews	Were repeat interviews carried out? If yes, how many?	No	Page 4
19. Audio/visual recording	Did the research use audio or visual recording to collect the data?	Yes (Zoom, WhatsApp audio/video recorded).	Page 3
20. Field notes	Were field notes made during and/or after the interview or focus group?	Yes – notes and reflections were systematically collected during and immediately after interviews.	Page 3
21. Duration	What was the duration of the interviews or focus group?	45–60 minutes.	Page 3
22. Data saturation	Was data saturation discussed?	Yes	Page 2
23. Transcripts returned	Were transcripts returned to participants for comment and/or correction?	No	Page 6
**Domain 3: analysis and findings**			
Data analysis			
24. Number of data coders	How many data coders coded the data?	All coding was undertaken by AS; reviewed with HE and SG.	Page 4
25. Description of the coding tree	Did authors provide a description of the coding tree?	Themes and sub-themes are presented in Table 3.	Page 6
26. Derivation of themes	Were themes identified in advance or derived from the data?	Inductively derived from the data.	Page 4, 6
27. Software	What software, if applicable, was used to manage the data?	Microsoft Word for manual coding.	Page 4
28.Participant checking	Did participants provide feedback on the findings?	No	Page 6
Reporting			
29. Quotations presented	Were participant quotations presented to illustrate the themes / findings? Was each quotation identidied? E.g. participant number	Yes – throughout Results section. Each quotation identified with participant number, age, gender, and faith tradition.	Page 7 – 11
30. Data and findings consistent	Was there consistency between the data presented and the findings?	Yes – the research team reviewed the thematic structure to ensure findings were consistent with raw data.	Page 4
31. Clarity of major themes	Were major themes clearly presented in the findings?	Yes – four major themes presented.	Page 7 – 11

32. Clarity of minor themes	Is there a description of diverse cases or discussion of minor themes?	Yes – descriptive sub-themes presented in Section 3.	Page 7 – 11

Developed from: Tong A, Sainsbury P, Craig J. Consolidated criteria for reporting qualitative research (COREQ): a 32-item checklist for interviews and focus groups. *International Journal for Quality in Health Care*. 2007. Volume 19, Number 6: pp. 349 – 357
