# Supplementary material for: The perspectives of Lagos faith-institution leaders on health, hypertension, and faith-based hypertension interventions: a qualitative interview study
Source: Front Public Health. 2026 Sep 9;14:1925205. doi: 10.3389/fpubh.2026.1925205 (PMC13597788; doi:10.3389/fpubh.2026.1925205)
Supplement: Supplementary file 2 [file Data_Sheet_2.PDF]

Supplementary File 2 – Codebook

Codebook Summary: Thematic Analysis of Faith Leaders' Perspectives on Hypertension

This document provides a summary of the hierarchical coding structure developed during the inductive thematic analysis. It illustrates the progression from initial open codes to closed codes, and finally to the descriptive and analytical themes presented in the main manuscript. The analysis remained grounded in the data, with codes and themes derived iteratively through constant comparison (as described in Section 2.7).

Note: The colour gradient (dark to light) indicates the level of abstraction: Analytical Themes (darkest), Descriptive Themes, Closed Codes, Sample Open Codes (lightest). Example quotations are left uncoloured to highlight the raw data.

Analytical Theme	Descriptive Theme	Closed Code	Open Code (Examples)	Example Quotation
**Deficient healthcare system**	The current state	Healthcare infrastructure inadequacy	• "shambolic healthcare situation"<br>• "outdated infrastructure"<br>• "poor awareness"	*"The healthcare situation is shambolic. Death because of hypertension continues because of poor awareness and poor belief."* (Respondent 3, Middle Aged Male ATR priest)
	Fundamental shortcomings	Governance and resource failures	• "questionable governance"<br>• "inadequate resources"<br>• "absence of functional primary healthcare"	*"The citizens don't seem to have that confidence in a government that their future is secure."* (Respondent 2, Middle Aged Male Pentecostal Christianity Pastor)
	Reality	Socioeconomic constraints	• "widespread unemployment"<br>• "inadequate health promotion"<br>• "non-existent health surveillance"	*"If they made it free, people will have access to it. If someone is not down they will not attend the hospital."* (Respondent 1, Middle aged male Sunni

Analytical Theme	Descriptive Theme	Closed Code	Open Code (Examples)	Example Quotation
				Imam)
**Severely impacted society**	Defective understanding	Health literacy gaps	• "ignorance about hypertension"<br>• "superstitious beliefs"<br>• "hypertension diagnosed late"	*"Hypertension is a bodily problem. A physical problem, not a spiritual problem."* (Respondent 3, Middle Aged Male ATR priest)
	Hierarchy of needs	Poverty-driven health decisions	• "prioritising daily needs over healthcare"<br>• "high cost of healthcare"<br>• "poverty forces suboptimal decisions"	*"The first thing is meal. They don't care until maybe the illness gets to a level where it is very severe."* (Respondent 1, Middle aged male Sunni Imam)
	Severe social impact	Preventable morbidity and mortality	• "preventable deaths"<br>• "decimating human resources"<br>• "premature mortality"	*"Hypertension has a high impact on the society by decimating the human resources and cutting lives short."* (Respondent 5, Male Ahmadi Islam Imam under 50 years old)
	Unavoidable responsibility	Shared accountability	• "collective responsibility"<br>• "government, NGOs, and faith institutions"<br>• "everybody in society has responsibilities"	*"Everybody in the society has responsibilities including NGOs as well as religious and Faith-leaders like myself."* (Respondent 3, Middle Aged Male ATR priest)
	Potential	Collaborative	• "integrating poverty alleviation into	*"For example using the media, like radio programmes. A*

Analytical Theme	Descriptive Theme	Closed Code	Open Code (Examples)	Example Quotation
	benefits	solutions	campaigns"<br>• "mass media engagement"<br>• "innovative solutions"	*large number of people can be reached..."* (Respondent 6, Female ATR Priestess under 50 years old)
**Underutilised faith institutions**	Unrewarding effort	Health ministry limitations	• "frustration about limitations"<br>• "lack of innovative solutions"<br>• "inadequate healthcare provision"	*"We can't compel individuals to attend. But those who know the importance of these health checks do attend these tests."* (Respondent 1, Middle aged male Sunni Imam)
	Potential for optimism and collaboration	Mixed optimism and resignation	• "health ministry efforts encouraging"<br>• "absence of collaborations"<br>• "optimism that viable solutions could be found"	*"Let's start working toward this direction and let's see how the country at large can be set free from the hypertension..."* (Respondent 7, Male Judaism Rabbi under 50 years old)
	Capable assets for innovation	Unique institutional capabilities	• "grassroots community connections"<br>• "tailor campaigns to religious faith"<br>• "enthusiasm for collaboration"	*"The church is a care organisation: whatever the church can do to help people manage their blood pressure, we'll be favourably disposed."* (Respondent 4, Middle Aged Male Pentecostal Christianity Pastor)
	Advocates for multi-institution collaboration	Government accountability and leadership	• "limited resources of faith institutions"<br>• "government uniquely placed to harness capabilities"<br>• "government must accept assistance"	*"The best way … is for the government to prioritise hypertension, the religious organisations to prioritise it."* (Respondent 5, Male Ahmadi Islam Imam under 50 years old)

Analytical Theme	Descriptive Theme	Closed Code	Open Code (Examples)	Example Quotation
**Possible change of approach**	Advocates for change	Transparency and responsiveness	• "greater transparency in resource allocation"<br>• "heightened responsiveness to evolving needs"<br>• "training and deploying traditional practitioners"	*"We've given support to the state government in terms of having a greater Lagos."* (Respondent 5, Male Ahmadi Islam Imam under 50 years old)
	Non-governmental solutions are possible	Cross-sector collaboration	• "exclusive reliance on health system unrealistic"<br>• "faith settings and local communities"<br>• "collaborations across congregations"	*"If the churches and mosques in collaboration with NGOs can team up together..."* (Respondent 2, Middle Aged Male Pentecostal Christianity Pastor)
	Resources and leadership drive change	Grassroots healthcare delivery	• "most effective healthcare delivered at grassroots"<br>• "locally generated knowledge"<br>• "financial resources exceed institutional means"	*"Getting healthcare at the grassroots level so that there won't be the need to travel far to get healthcare."* (Respondent 7, Male Judaism Rabbi under 50 years old)
	Solutions are possible through faith institution influence	Integration into health ministries	• "health ministries already provide health promotion"<br>• "opportunities for innovative strategies"<br>• "adaptation of religious culture and doctrines"	*"Because in Nigeria most of the population believe in God, Mosques are full. Churches are also full up... Government can target these areas."* (Respondent 1, Middle aged male Sunni Imam)
